# Nano‐Coating Loaded With Leaf and Flowers of *Pelargonium graveolens* Plant Extract Stabilized With Fenugreek Seed Gum and Soy Protein Isolate in Increasing the Shelf Life of Mutton Fillet

**DOI:** 10.1002/fsn3.4618

**Published:** 2024-12-30

**Authors:** Farzad Ebrahimi, Nader Habibi, Mohammadyar Hosseini

**Affiliations:** ^1^ Department of Food Science and Technology, Sanandaj Branch Islamic Azad University Sanandaj Iran; ^2^ Department of Food Science and Hygiene, Faculty of Veterinary Science Ilam University Ilam Iran

**Keywords:** antibacterial, antioxidant, edible coating, emulsion, meat

## Abstract

In this study, the extract of leaf and flower of *Pelargonium graveolens* was obtained using an ultrasonic‐assisted extraction method. The extraction yield and the content of phenolic, flavonoid, and flavonol compounds in the flower extract were higher (13.93%, 74.97 mg GAE g DM^−1^, 31.93 mg QE g DM^−1^, and 9.08 mg QEE g DM^−1^) than leaf extract (10.69%, 67.46 mg GAE g DM^−1^, 23.04 mg QE g DM^−1^, and 11.34 mg QEE g DM^−1^). Both extracts demonstrated antioxidant properties in tests involving the scavenging of DPPH radicals and the ferric reduction assay. Extracts exhibited antimicrobial properties. MIC of flower extract against *Staphylococcus aureus* and *Escherichia coli* were 2500 and 5000, while MBC of leaf extract were 15,000, and 20,000 ppm, respectively. The concentration of 2000 ppm of extracts was encapsulated in fenugreek seed gum (FSG) and soy protein isolate (SPI) produced by the emulsification method. All nano‐coatings exhibited a nanometric size range between 172.75 to 255.21 nm, and encapsulation efficiency higher than 80.0% (80.82% to 89.59%). The application of nano‐coatings significantly reduced microbial counts and delayed lipid oxidation in mutton meat during 12 days of cold storage at 4°C, enhancing meat quality and extending shelf life. The inclusion of bioactive compounds like polyphenols in the coatings contributed to antimicrobial and antioxidant effects, decreasing pH levels and preventing spoilage. The findings indicated that the combination of edible FSG and SPI as wall materials with 2000 ppm of *P. graveolens* extract demonstrated efficacy in implementation bacterial growth and lipid oxidation in fresh mutton meat.

## Graphical abstract

In this paper, the extract of *Pelargonium graveolens* plant was obtained from flowers and leaves. Antioxidant and antimicrobial properties of extracts were evaluated in the in vitro conditions. The results confirm the higher antioxidant and antimicrobial activity of flower extract and these potentials can make the extract a good alternative to synthetic preservatives. Therefore, the extract nanoencapsulated in the soy protein isolates and fenugreek seed gum as natural biopolymers. This coating applied on mutton meat could reduce microbial growth and lipid oxidation. The results of this study can be used in meat industries.

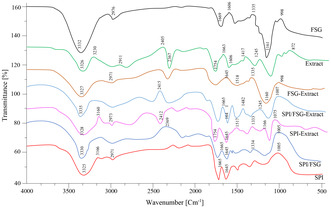

## 1 Introduction

Mutton, a widely consumed red meat, is an important dietary component due to its rich nutrient profile (González et al. 2020; Tsitsos et al. 2021). It provides high‐quality proteins, essential amino acids, and a variety of B vitamins, including B_6_, B_12_, niacin, riboflavin, and pantothenic acid (Pethick et al. 2021). Furthermore, mutton contains key minerals such as phosphorus, iron, selenium, and zinc, along with polyunsaturated fatty acids like omega‐3 (Tsitsos et al. 2023).

Meat, due to its high moisture, protein, and fat content, is prone to spoilage, bacterial contamination, and lipid oxidation, posing health risks if not properly handled and cooked (Economou et al. 2022; Song et al. 2020; Yuan, Chen, and Li 2016). It can serve as a vector for foodborne pathogens, notably *Staphylococcus aureus* and *Escherichia coli* , which are commonly found in both humans and domestic animals and are known to cause human diseases (Bennett, Walsh, and Gould 2013; Economou et al. 2022).

To counteract the growth of microorganisms and prevent oxidation, as well as extend the storage duration of red meat, the utilization of various food additives is commonly practiced. Nevertheless, the prevailing attitudes of certain consumers play a significant role in the shift toward substituting synthetic food additives with natural alternatives (Carrapiso et al. 2023; Odeyemi et al. 2020).

Plants are recognized as valuable sources of natural antioxidant and antimicrobial agents, with certain species offering extracts rich in phytochemicals with health benefits. These effects are primarily attributed to bioactive compounds such as flavonoids, phenolic acids, tannins, and phenolic diterpenes, with flavonoids and polyphenols receiving notable attention for their health impacts (Ebrahimian, Najafi, and Abedinia 2024; Esmaeilzadeh Kenari and Razavi 2022; Gorzin et al. 2024; Razavi and Kenari 2021; Razavi et al. 2021).

Research on bioactive compounds in plants has highlighted their potential in promoting health, with studies underscoring the significant contributions of these compounds to antioxidant, antimicrobial, and medicinal activities (Dimitrova et al. 2015; Razavi and Kenari 2021). Rose geranium ( *Pelargonium graveolens* ), a member of the Geraniaceae family, is a valuable medicinal herb that has been used in traditional medicine for centuries. The plant's infusions are recognized for their health benefits, and even today, *P. graveolens* extracts are used as food flavors, demonstrating its continued importance (Dimitrova et al. 2015; Ennaifer et al. 2020).

The antioxidant and antimicrobial properties of *graveolens* extracts (GE) have gained significant attention for their health benefits. These extracts have been linked to the treatment of various conditions, including wound healing, fatigue, gastrointestinal disorders, excessive bleeding, skin issues, nerve pain, and throat infections, all of which impact well‐being (Abdelbaky et al. 2022; Dimitrova et al. 2015; Ennaifer et al. 2020).

Edible coatings, thin films applied to various food products, aim to enhance quality and extend shelf life. These coatings, often used as carriers for preservative extracts, improve the overall attributes of food, keeping them fresh and appealing for longer periods (Guo et al. 2024; Song et al. 2021). Natural polymers, such FSG, offer advantages over synthetic alternatives, being cost‐effective, biodegradable, biocompatible, and environmentally friendly (Shukla et al. 2020). FSG, derived from *Trigonella foenum‐graecum* seeds (Fatemi et al. 2024), has been widely used in various applications like drug release retardation, binding, emulsification, gelling, and film formation (Shukla et al. 2020).

Soy protein is derived from soybeans, which are primarily used for extracting soy oil. In the extraction process, soy flour is produced as a by‐product, which can further undergo purification to yield concentrate and SPI, thereby enhancing the value of agricultural residues. Moreover, soy proteins exhibit the capability to create films and coatings with exceptional functional characteristics, including outstanding oxygen barrier properties (Guerrero et al. 2015; Xu et al. 2024).

Nanotechnology, a rapidly advancing field, has the potential to revolutionize numerous sectors, positioning itself as a key industrial development of the century (Dash et al. 2020; Shukla et al. 2020). Its applications span industries such as food, chemistry, agriculture, biology, and medicine (Khan, Saeed, and Khan 2019; Patil and Kim 2017). Nanomaterials exhibit superior physicochemical and biological properties compared to bulk materials (Razavi et al. 2020), making them promising for various scientific applications (Nilavukkarasi, Vijayakumar, and Prathipkumar 2020). Nanoparticles, with their high surface‐to‐volume ratio, are ideal for performance‐based uses such as encapsulation and edible packaging, facilitating their integration into commercial, technological, and food products (Abdelbaky et al. 2022; Xu et al. 2020).

This study marks the first instance of using *P. graveolens* extract in nano‐coating form combined with SPI and FSG to extend the shelf life of meat. While edible coatings have been explored for food preservation, the application of this plant extract encapsulated in nano‐coatings has not been previously reported. The findings of this research offer potential for producing meat products with extended shelf life without relying on synthetic additives, thereby addressing consumer demand for more natural and healthier alternatives.

## 2 Materials and Methods

### 2.1 Materials

A mutton carcass (Moisture = 69.5%, Protein = 20.6%, Fat = 8.9%, Ash = 1%) was supplied from the local market (Protein Plus, Sari, Mazandaran, Iran). SPI (Protein content ≥ 90%) was acquired from Shansong Biological Products Co. Ltd. (Linyi, Shandong, China). *S. aureus* and *E. coli* were provided by the Iranian Research Organization for Science and Technology (IROST, Tehran, Iran). All chemicals were used in analytical grade and were obtained from the Scharlau (Barcelona, Spain). Fenugreek seeds were kindly obtained from grocery. The geranium plant was obtained from the Karaj Agricultural Research Center (Karaj, Iran), and has been identified on the basis of its morphological and botanical characteristics.

### 2.2 Methods

#### 2.2.1 Plant Extraction

The air‐dried leaf and flower of *P. graveolens* were subjected to the ultrasonic‐assisted solvent extraction method. To begin, 20 g of the resulting powder was combined with 400 mL of deionized water. The extraction process took place in a conical flask, which was then exposed to ultrasonic waves using a Probe sonicator (VC50, Danbury, USA, 150 W, 25 kHz) at room temperature (23 ± 2°C) for a duration of 30 min. After the extraction, the mixture was filtered through muslin cloth and Whatman filter paper No.1 to separate the solvent and powder. The resulting filtrate solution of extract was then stored in a refrigerator for future use (Abdelbaky et al. 2022). The extraction yield of the extract was determined gravimetrically (Kenari and Razavi 2022).

#### 2.2.2 FSG Extraction

Fenugreek seeds were cleaned by washing them with water and then roughly ground using a grinder (P320, Pars‐Khazar, Iran). Subsequently, the powder was soaked in distilled water for a duration of 10 h, following which the gum was separated from the mixture by filtration through muslin cloth. The resulting filtrate underwent precipitation with ethanol on multiple occasions to ensure thorough extraction. Finally, the gum was dried in the oven at 60°C, powdered, and stored in a polythene container for future utilization (Shukla et al. 2020).

#### 2.2.3 Total Phenolics, Flavonoids, and Flavonols Measurement

To evaluate the total phenolic content of GE, 0.5 mL of diluted extract (10% w/v) or a standard phenolic compound (Gallic acid) was combined with 5 mL Folin–Ciocalteu reagent (10% w/v) and 4 mL aqueous Na_2_CO_3_ 1 M. The quantification of total phenolic content was carried out using a UV spectrophotometer (UV‐160A, Shimadzu, Kyoto, Japan). To measurement the total flavonoids content 0.5 mL of extract was mixed with 4.5 mL distilled water, 0.3 mL of 5% NaNO_2_, 1 mL of 10% AlCl_3_·6H_2_O after 6 min, and 2 mL of 1 M NaOH and distilled water after another 5 min to achieve a final volume of 10 mL. The absorbance was promptly recorded at 510 nm using a spectrophotometer (Amiri, Nikbakht, and Etemadi 2015). To assess flavonol content, a standard curve of rutin through the combination of 2 mL of various concentrations of methanolic rutin solutions with 2 mL of AlCl_3_ (20 mg mL^−1^) and 6 mL of sodium acetate (50 mg mL^−1^) was used. Subsequently, the absorbance was recorded at 440 nm after a 2.5 h incubation period. The same experimental procedure was applied to 2 mL of plant extract (Boukhris et al. 2013).

#### 2.2.4 Antioxidant Activity

The antioxidant activity of leaf and flower extracts of *P. graveolens* was evaluated using 2, 2, diphenyl‐picryl‐hydrazyl (DPPH), and ferric reduction antioxidant power (FRAP) assays. The ability of the extracts to donate an electron DPPH radical was assessed by mixing a freshly methanolic solution of DPPH (4 × 10^−4^ M) with the leaf and flower extracts and a standard solution in a ratio of 2:0.5 (v/v). The light absorption was then measured at 515 nm. The DPPH radical scavenging activity was quantified based on the concentration of gallic acid using the following equation:

(1) $$\text{Inhibition}\;\left(\%\right)=\frac{A_{1}-A_{2}}{A_{1}}\times 100$$

where *A*_1_ and *A*_2_ are absorbance of the control without extract and absorbance of the extracts with DPPH radical.

The FRAP assay involved measuring the change in absorbance at 593 nm due to the formation of a blue‐colored Fe (II)‐tripyridyltriazine compound from colorless oxidized Fe (III) form by the action of electron‐donating antioxidants. The FRAP reagent was prepared from specific components in defined proportions, and the reaction mixture was recorded after allowing the plant extracts to react with the FRAP reagent solution for a specific duration at a controlled temperature (Dimitrova et al. 2015; Hajian‐Tilaki et al. 2024).

#### 2.2.5 Minimum Inhibitory Concentration (MIC) and Minimum Bactericidal Concentration (MBC)

The National Committee for Clinical Laboratory Standards (NCCLS) recommended the use of the broth dilution method to determine the MIC and MBC. The experiment was conducted using sterile 96‐well microplates. The samples were suitably prepared and distributed into individual wells of the microplate in order to establish a sequential dilution of the original extract. To promote consistent diffusion, the extracts were prepared in 0.1% ethanol as stock solutions. Each well received an inoculum containing 10^4^ colony‐forming units (CFU) of bacteria, except for the negative control wells which contained only bacteria in their respective medium. After that, 30 mL of 0.02% resazurin and 12.5 mL of 20% Tween 80 were added. The plates were then incubated aerobically at 30°C for 16–20 h. Following incubation, the wells were observed for a color change from blue to pink, indicating bacterial growth inhibition. The MIC was defined as the lowest concentration of the extracts that inhibited bacterial growth. To determine the MBC, broth from each tube was sub‐cultured for 24 h in tryptic soy broth for bacteria (Boukhris et al. 2013).

#### 2.2.6 Extract Encapsulation

The resulting mixture was subsequently cooled and stored at a temperature of 4°C overnight to boost the process of hydration. Then, 40 mL of Tween 80 (with a hydrophilic–lipophilic balance value of 15) emulsifier and 50 mL of sunflower oil were combined with 10 mL of different extract. This mixture was then subjected to mixing in an Ultra‐Turrax homogenizer (T‐25 basic, IKA, Germany) at a speed of 12,000 rpm for a duration of 5 min, resulting in the formation of an emulsion. The final emulsion was subsequently encapsulated with various coating materials at a ratio of 2:5. To further decrease the size of the particles, a probe‐type ultrasound device (VC50, Danbury, USA) with a power of 150 W and a frequency of 25 kHz was utilized for a duration of 5 min (Sarvinehbaghi et al. 2021).

#### 2.2.7 Characteristics of Nano‐Coating

The Z‐average diameter, polydispersity index (PDI), and zeta potential of the nano‐coating samples were determined utilizing the Zetasizer (ZS90, Malvern, UK) through laser light scattering methodology. The samples underwent a 10‐fold dilution with deionized water before being introduced into the tube. The encapsulation efficiency (EE) was computed using Equation (2):

(2) $$\mathrm{EE}\;\left(\%\right)=\frac{P1-P2}{P1}\times 100$$

where P1 = Total phenolic compounds, and P2 = Surface compounds of sample.

The morphology of the nano‐coating was examined using a scanning electron microscope (SEM) (S4800, Hitachi, Japan). The samples were coated with a thin layer of gold and then transferred to a vacuum evaporator. Subsequently, a beam of high‐velocity electrons with an accelerated voltage of 26 kV was directed toward the samples to generate scanned images for analysis (Jafari et al. 2022).

Infrared analyses were conducted using a Fourier‐transform infrared spectroscopy infrared (FTIR) (1600 series, Perkin Elmer Co. Norwalk, USA) equipped with a horizontal attenuated total reflectance (ATR) accessory. The spectra were collected in the wavenumber region of 500–4000 cm^−1^ using 60 scans for each sample. Prior to sample analysis, a background spectrum was recorded with the crystal free of any sample. Sample spectra were acquired by applying the sample onto the ATR crystal surface and recording the spectrum (Oliveira et al. 2022).

The crystalline structure of the nano‐coating with and without extract was evaluated using X‐ray (PW1730, Philips, the Netherlands) diffraction analysis (XRD) according to the method described by Shukla et al. (2020). The X‐ray diffraction grams were run at a scanning speed of 2° min^−1^ and chart speed of 2°2 cm^−1^ per 2. All samples were dried, ground, and then measured (Shukla et al. 2020).

#### 2.2.8 Meat Coating and Storage

Firstly, the meat was carefully washed with sterile distilled water. Then, it was precisely divided into uniform segments 2 × 2 × 2 cm under strict aseptic conditions using a sterile razor. Fresh mutton meat pieces were then immersed in specially prepared coating solutions for 1 min. Subsequently, the coated meat pieces were dried for 1 min at room temperature under a laminar airflow to prevent any potential contamination. The mutton meat samples were enclosed in sterile zippered plastic bags and stored at 4°C for 12 days. The selection of this storage time was based on our prior treatments. In order to assess the efficacy of the coatings during the cold storage period, the total viable count of bacteria, pH, and lipid oxidation of each sample were analyzed at various time points, including immediately after production and at 2, 4, 6, 8, 10, and 12 days of storage (Jooyandeh et al. 2023).

#### 2.2.9 Quality Assessment of Meat Samples

The meat's total viable count (TVC) was examined using the methodology outlined by Song et al. (2020). To estimate the TVC, plate count agar was utilized. Initially, a sterile homogeneous bag containing 45 mL of NaCl 0.85% solution without bacteria was prepared. Subsequently, 5 g of the meat sample was transferred aseptically into the bag and homogenized for a duration of 1 min. From this initial dilution, additional decimal dilutions were prepared using NaCl solution. Each appropriate gradient dilution was then carefully pipetted onto the surface of plate count agar. Following this, the inoculated plates were incubated for a period of 72 h at a temperature of 30°C to allow for TVC growth (Song et al. 2020).

A total of 10 g mutton meat was blended with sterile distilled water in a 1:10 ratio, followed by a homogenizer operating at 10,000 rpm for a duration of 1 min (T10, IKA, Germany). The freshly prepared homogenate was submitted to a digital pH meter (713 CH‐9101, Metrohm, Herisau, Switzerland). Prior to measurement, the pH meter underwent calibration with buffer solutions at pH values of 4.0, 7.0, and 9.0 at a temperature of 25°C (Zhao et al. 2022).

Lipid oxidation was assessed through the formation of thiobarbituric acid reactive substances (TBA). To conduct the analysis, 10 g of meat samples underwent homogenization with 35 mL trichloroacetic acid (5%, w/v) using a homogenizer at 4500 rpm and 5°C for 1 min, followed by centrifugation at 2500 rpm and 5°C for 20 min and filtration of the supernatant through Whatman filter paper No. 1. During the homogenization process, the test tubes were maintained in an ice bath at 5°C to minimize the occurrence of oxidative reactions while extracting TBA. Subsequently, a mixture of 2 mL of TBA solution and 2 mL of the filtered supernatant was heated in a water bath (80°C) for 20 min. After cooling at 10°C, the absorbance was measured at 532 nm using a spectrophotometer (Langroodi et al. 2018).

### 2.3 Statistical Analysis

The experiments were conducted in triplicate (*n* = 3) for each measurement. The data obtained were then presented as the mean value ± standard deviation. Analysis of the data was conducted using ANOVA, with significant differences being evaluated through Duncan's multiple range test or t‐student test (*p* < 0.05). The figures were generated using the Excel 2020 program.

## 3 Results and Discussion

### 3.1 Extraction Yield

The extraction yield from flowers and leaves of GE obtained with water is shown in Table 1. Flowers extract showed a higher extraction yield than leaf extract, and significant statistical differences (*p* < 0.05) were observed. In a study conducted by Harzallah, Hachama, and Khadraoui (2022), the extraction yield of *P. graveolens* obtained by petroleum ether, chloroform, butanol, and ethanol were 7.20%, 8.80%, 31.12%, and 33.60%, respectively (Harzallah, Hachama, and Khadraoui 2022). Ahmed et al. (2011) conducted an experiment where they utilized methanol, acetone, and ethanol solvents to extract the leaf and flower extract of the Akk ( *Calotropis procera* ) plant. Their findings revealed that the extraction yield of the flower was superior to that of the leaf (Ahmad et al. 2011).

**TABLE 1:** Extraction yield, the content of phenolic, flavonoid, and flavonol compounds of flower and leaf of *Pelargonium graveolens* extract.

Extract	Extraction yield (g 100 g DW^−1^)	Total phenolic (mg GAE g DM^−1^)	Total flavonoid (mg QE g DM^−1^)	Total flavonols (mg QEE g DM^−1^)
Flower	13.93 ± 1.58^a^	74.97 ± 2.43^a^	31.93 ± 1.77^a^	9.08 ± 0.38^b^
Leaf	10.69 ± 0.99^b^	67.46 ± 1.91^b^	23.04 ± 1.88^b^	11.34 ± 0.76^a^

*Note:* The results are presented as the mean ± standard deviation of three determinations. Means that are denoted by different letters showed significant differences (*p* < 0.05).

### 3.2 Total Phenolic, Flavonoid, and Flavonol Content

Plant phenolic compounds have garnered increasing attention as valuable components for functional foods and nutraceutical products due to their potential positive impacts on health. Phenolic compounds found in plants are recognized for their strong natural antioxidant properties, which can be beneficial for human health. Numerous research studies have highlighted the significant role of total phenols and flavonoids in the antioxidant capabilities of various plants (Ahmad et al. 2011; Kenari and Razavi 2022; Razavi and Kenari 2021). For instance, investigations have shown that the antioxidant activities of plants are closely linked to the levels of total phenolics and total flavonoids present in them (Harzallah, Hachama, and Khadraoui 2022). In the case of *P. graveolens* , the extracts obtained from its leaf and flowers contain varying amounts of total phenolics, flavonoids, and flavonols, as indicated in Table 1. The levels of total phenolics were 74.97 and 67.46 mg GAE g DM^−1^, while the total flavonoid content was 31.93 and 23.04 mg QE g DM^−1^ for flowers and leaf extract, respectively. These findings underscore the potential health benefits that can be derived from incorporating plant phenolics into dietary supplements and functional foods. Higher content of phenolic and flavonoid compounds in the flowers extract than leaf extract was also reported by other researchers for *Calotropis procera* (Ahmad et al. 2011), *P. graveolens* (Boukhris et al. 2013; Harzallah, Hachama, and Khadraoui 2022). The value acquired in this study was found to be lower than the reported findings by Ahmed et al. (2011) regarding the levels of phenolic, and compounds in *P. graveolens* leaf extract. This disparity may be attributed to the type of solvent, the utilization of ultrasound as the extraction method, as well as the influence of the plant's growth environment on its phytochemical constituents (Razavi and Kenari 2021).

### 3.3 Antioxidant Activity

**FIGURE 1:**
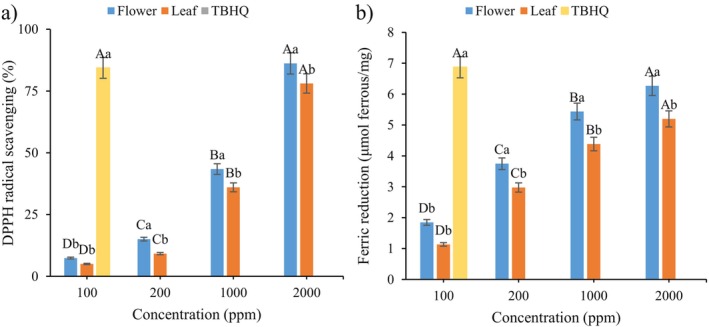
(a) DPPH radical scavenging and (b) ferric reduction activity of flower and leaf *Pelargonium graveolens* extract. Different uppercase letters indicate a statistically significant difference (*p* < 0.05) between different concentrations in the same extract. Different lower‐case letters indicate a statistically significant difference (*p* < 0.05) between different extracts at the same concentration.

In this study, DPPH and FRAP assays were subjected to evaluate the antioxidant activity of extracts. DPPH encounters a hydrogen donor, particularly phenolics, it undergoes a transformation where its chromophore is lost, resulting in a yellow coloration. Figure 1 shows the results of DPPH radical scavenging and ferric reduction activity of different extracts. The antioxidant activity in both methods demonstrated an upward trend as the extract concentrations were increased. Flowers extracts exhibited higher antioxidant activity than leaf extracts. Lower half‐maximal inhibitory concentration (IC50) for methanol and aqueous of *P. graveolens* flower was reported (Boukhris et al. 2013). Ennaifer et al. (2020) examined the antioxidant characteristics of aqueous extracts from *P. graveolens* by analyzing their ability to scavenge free radicals using the DPPH method and conducting a b‐carotene bleaching test. The results indicated a significant radical scavenging activity exhibited by the extract (Ennaifer et al. 2020). It is widely acknowledged that the scavenging activity of DPPH radical, as well as the antioxidant potential of a plant extract or a similar compound, is enhanced with an increase in the concentration of phenolic compounds or the degree of hydroxylation of these compounds (Dimitrova et al. 2015; Harzallah, Hachama, and Khadraoui 2022; Razavi and Kenari 2021). The observed data did not reveal any statistically significant difference (*p* > 0.05) when comparing tert‐butylhydroquinone (TBHQ) with 2000 ppm of each extract. Therefore, this concentration was used for encapsulation.

### 3.4 Antimicrobial Activity

Table 2 presents the MIC and MBC of the extracts of *P. graveolens* against the microorganisms tested. The findings indicate that the extracts possess bactericidal properties, with the concentrations needed to kill the bacteria generally being twice as high as the concentrations required to inhibit their growth, as indicated by the respective MBC and MIC values. Notably, the flower extract exhibited greater potency compared to the leaf extract. Previous studies have suggested the bactericidal influence of phenolic and flavonoid compounds present in extracts (Boukhris et al. 2013; Harzallah, Hachama, and Khadraoui 2022).

**TABLE 2:** MIC and MBC of flower and leaf extract of *Pelargonium graveolens* extract (ppm).

Extract	*Staphylococcus aureus* (ATCC 25923)		*Escherichia coli* (ATCC 25922)	
	MIC	MBC	MIC	MBC
Flower	2500	5000	5000	10,000
Leaf	7500	15,000	10,000	20,000

*Note:* The results are presented as the mean ± standard deviation of three determinations. Means that are denoted by different letters showed significant differences (*p* < 0.05).

### 3.5 Properties of Nano‐Coating

The properties of nano‐coatings in terms of average particle size, polydispersity index (PDI), zeta potential, and encapsulation efficiency are presented in Table 3. Particle size is a crucial parameter when considering the utility of the encapsulated materials (Razavi et al. 2020). The findings indicated that nano‐coating loaded with extracts and stabilized with SPI/FSG exhibited a smaller particle size compared to those formulated with either SPI or FSG alone. This suggests that the presence of FSG in the coating might have minimized protein nanoparticle aggregation by promoting electrostatic repulsion between them. Research has demonstrated that smaller particle sizes play a significant role in enhancing the application efficiency of the encapsulated active ingredients (Ahmadian et al. 2024).

**TABLE 3:** *Z*‐average diameter, PDI, zeta potential, and encapsulation efficiency of nano‐capsules stabilized by SPI and FSG.

Sample	*Z*‐average diameter (nm)	PDI	Zeta potential (mV)	Encapsulation efficiency (%)
SPIF	244.61 ± 10.09^a^	0.152^a^	27.59 ± 2.58^d^	80.82 ± 0.3.38^c^
SPIL	255.21 ± 16.31^a^	0.159^a^	29.40 ± 1.52^c^	82.89 ± 3.66^c^
FSGF	193.44 ± 5.38^b^	0.120^b^	31.70 ± 2.15^b^	84.92 ± 4.01^b^
FSGL	185.62 ± 4.44^b^	0.116^bc^	31.88 ± 0.84^b^	85.57 ± 2.34^b^
MIXF	172.75 ± 9.72^c^	0.107^d^	32.93 ± 0.88^ab^	88.06 ± 3.03^a^
MIXL	178.79 ± 4.77^c^	0.111^cd^	33.43 ± 1.57^a^	89.59 ± 3.29^a^

*Note:* The results are presented as the mean ± standard deviation of three determinations. Means that are denoted by different letters showed significant differences (*p* < 0.05). SPIF and SPIL = flower and leaf extract stabilized with soy protein isolate. FSGF and FSGL = flower and leaf extract stabilized with Fenugreek seed gum. MIXF and MIXL = flower and leaf extract stabilized with composition of soy protein isolate and Fenugreek seed gum.

The PDI serves as an indicator of both particle uniformity and stability within a sample. A higher PDI value is associated with samples containing a wider range of particle sizes, while samples with particles of uniform size exhibit lower PDI values. Therefore, the PDI value is utilized to quantify the distribution of particle sizes within a given sample. The PDI values of nano‐coatings with SPI and FSG wall materials individually, as well as a combination of SPI and FSG, ranged from 0.107 to 0.159. The findings suggested the existence of consistent and uniformly sized particles that are stable and monodisperse.

The z‐potential value serves as an indicator of the stability of complex formed using biopolymer coatings. The presence of electrostatic repulsion plays a crucial role in preventing the aggregation of complexes, particularly those with *z*‐potential values between −30 to +30 mV, thereby enhancing the stabilization of the corresponding emulsions as referenced. In the investigation conducted, the *z*‐potentials recorded for nano‐coatings that were stabilized using SPI, FSG, and SPI/FSG ranged from 27.59 to 33.43 mV. It was observed that the choice of hydrophilic emulsifier utilized during the emulsification process had a notable influence on the *z*‐potential values obtained. The negative *z*‐potential values detected in the prepared nano‐coating indicated the presence of negatively charged molecules. The carboxylic acid in seed gum structures contributes to the generation of a negative charge (Kenari and Razavi 2022).

The increase in *z*‐potential observed in nano‐coating with complex wall materials was attributed to the electrostatic interaction between positively charged fragments present in protein molecules and the anionic groups found in FSG. The results obtained suggest that the utilization of this complex can facilitate the production of food‐grade nano‐coatings. This highlights the potential for creating stable emulsions for various applications by leveraging the unique properties of these components in the emulsification process (Choi and Chang 2018). These results are in agreement with other researchers for encapsulated hyssop extract in chia seed gum and SPI (Ahmadian et al. 2024), gallic acid in whey protein isolate, and polysaccharide extracted from *Ulmus davidiana* (Choi and Chang 2018).

The encapsulation efficiency of the extract surpassed 80% across all wall materials, demonstrating the exceptional efficiency of the coating in entrapping the extract. The extract coated with the complex of protein and polysaccharide exhibited a superior encapsulation efficiency. It appears that the development of a strong protein‐polysaccharide complex has resulted in increased entrapment of the extract. These findings align with previous studies indicating that the encapsulation efficiency of the combined protein‐polysaccharide coating exceeds that of individual protein or polysaccharide coatings (Kenari et al. 2020).

**FIGURE 2:**
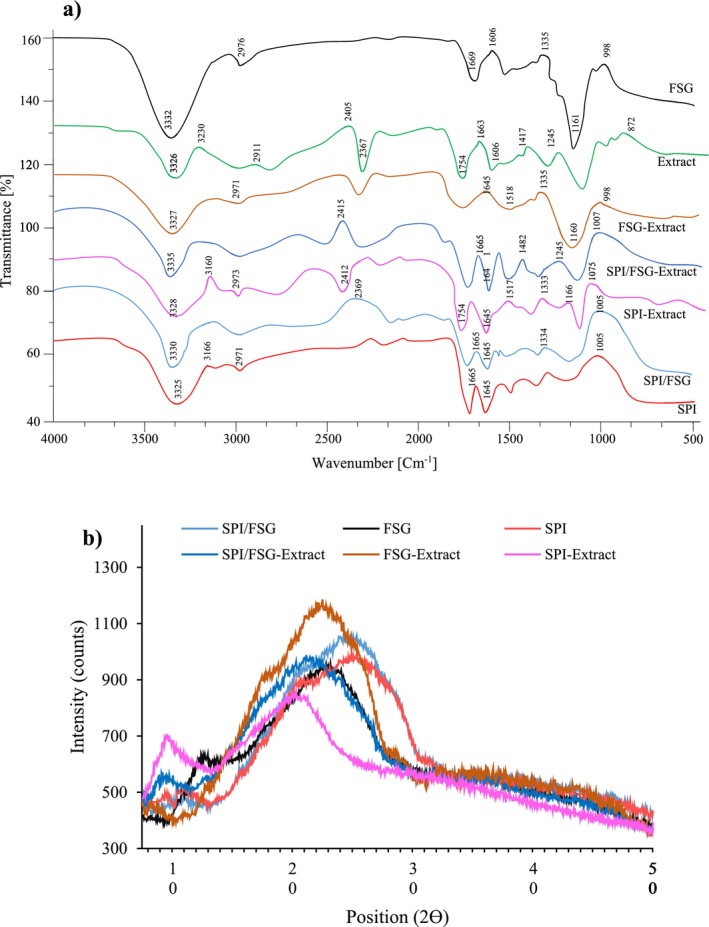
(a) FTIR spectra and (b) XRD patterns of fenugreek seed gum (FSG), soy protein isolate (SPI), a complex of FSG and SPI (MIX), *Pelargonium graveolens* extract (EXT), encapsulated *P. graveolens* flower extract in SPI (SPIF), FSG (FSGF), and a complex of FSG and SPI (MIXF).

Figure 2a illustrates the FTIR spectra of both individual and complex polymers, as well as the extract and encapsulated extract in polymers. This spectrum provides information about the functional groups present in these compounds based on the wavenumber (cm^−1^). Key peaks in the spectra correspond to specific vibrational modes of chemical bonds. In the 3200–3500 cm^−1^ range, a broad peak around 3300 cm^−1^ likely corresponds to O‐H or N‐H stretching vibrations, indicating the presence of hydroxyl groups, amines, or water. This peak is present in all samples but varies in intensity. The SPI exhibited peaks at 3325 cm^−1^ (OH and NH stretching vibrations) and 2971 cm^−1^ (CH stretching) (Ahmadian et al. 2024). Peaks around 2900 cm^−1^ in the 2800–3000 cm^−1^ range are associated with C‐H stretching, which typically indicates the presence of alkyl groups. The peaks observed at 1645 and 1517 cm^−1^ are linked to the CO and NH, respectively. Changes in the secondary structure of proteins are often associated with variations in the CO. Furthermore, the bands at 1482, 1333, and 1168 cm^−1^ indicate the CH bending vibration, CN stretching vibration, and amide III NH bending vibration, respectively (Ahmed, Fernández‐González, and García 2020).

The FTIR examination of the FSG revealed distinct peaks at 3327 cm^−1^ associated with OH band, 2911 cm^−1^ for CH, 1663 and 1482 cm^−1^ indicating COO of uronic acids, 1200 cm^−1^ signifying COC in the pyranose ring, 1075 cm^−1^ for COC of 1→4 glycosidic bonds and COH, and 872 cm^−1^ for β‐anomeric C–H deformation and glycosidic linkages of glucopyranose and xylopyranose units (Mansour, Salah, and Xu 2020; Timilsena et al. 2016). Around 1645 cm^−1^, peaks are likely due to C=O stretching vibrations from carbonyl groups or absorbed water, prominent in samples containing extracts and encapsulated compounds. Additionally, the 1540 cm^−1^ peaks are attributed to N‐H and C‐N vibrations, which are characteristic of amides, particularly evident in samples containing SPI. Peaks near 1450 cm^−1^ are related to C‐H bending vibrations, indicating aliphatic groups. In a study conducted by Kumar and Babu (2014) the FSG exhibited significant peaks at 1014.58 and 1641.54 cm^−1^, which is in line with our results. Also, they stated that the compatibility of the drug was confirmed as the gum was found to have no impact on the peaks (Kumar and Babu 2014).

In contrast, the SPI/FSG complex exhibited bands where the SPI functional groups were more dominant due to the higher protein concentration in the complex, resulting in reduced intensity compared to the individual SPI polymer. This decrease in intensity is likely due to an electrostatic interaction between FSG and SPI as suggested by (Tavares and Noreña 2020). The FTIR spectra of the SPI/FSG complex exhibited novel bands at 1162 and 2218 cm^−1^, signifying the existence of covalent bonds between proteins and polysaccharides. Additionally, Dong and Cui (2021) noted a considerable decrease in the intensity of the CO and NH bands of soy proteins and the phosphate group bands of gelatinized potato starch in complexes of soy proteins and gelatinized potato starch. This decrease emphasizes the connections between the phosphate groups of GPS and the amino groups of SPI, indicating the formation of a complex between the two constituents (Dong and Cui 2021).

Figure 2b illustrates the XRD patterns of both individual and complex polymers, as well as the extract and encapsulated extract in polymers. The XRD analysis highlights the distinct impacts of different encapsulation coatings on the structural properties of microencapsulated geranium leaf extract. FSG exhibits sharp, high‐intensity peaks, particularly around 2*θ*, indicating a semi‐crystalline structure attributed to its polysaccharide composition. Polysaccharides like those in FSG are known to form gel‐like, organized networks that enhance structural stability, which is crucial for protecting the bioactive compounds within the extract. This semi‐crystalline arrangement likely contributes to a more controlled release profile, as the structure stabilizes the encapsulated extract and mitigates premature degradation. In contrast, the SPI coating shows broader and lower‐intensity peaks, signifying a more amorphous and flexible structure. Proteins like those in SPI are inherently more disordered compared to polysaccharides, due to their molecular structure and weaker intermolecular interactions. The amorphous nature of SPI coatings can provide an adaptable matrix, beneficial for applications requiring gradual release; however, the lower structural organization may reduce the ability to maintain long‐term stability of the encapsulated compound. Thus, SPI's structure is more suitable for applications where flexibility and slower release are prioritized over structural integrity. The combination of FSG and SPI in the SPI/FSG coating demonstrates a balanced structure, with moderate peak intensity and width, combining the organizational stability of FSG with the flexibility of SPI. This hybrid structure is ideal for microencapsulation, as it achieves a balance between crystalline order and amorphous flexibility, optimizing both stability and release control. Such a balanced structure is likely to enhance the stability of the encapsulated geranium flower extract while allowing for controlled release. This outcome underscores the significance of coating selection in tailoring the encapsulation system's structural and functional properties to achieve specific release profiles and stability requirements for sensitive botanical extracts.

Al‐Shammari, Al‐Ali, and Al‐Sahi (2019) reported that FSG had one weak peak, which was observed at the scattering angle (20) and semi‐crystalline structure. In another study conducted by Shukla et al. (2020), XRD analysis of FSG reveals a rough surface with pores and crevices, characteristics that support its potential for sustained drug release. The irregular surface traps drug particles effectively, which enhances the gum's ability to control release rates. Additionally, FSG powder exhibits a wide particle size distribution, from large to ultrafine particles, contributing to a dense, closely packed structure. This arrangement, while beneficial for sustained release, may also lead to poor flow properties due to low porosity and compact packing (Shukla et al. 2020). Han, Yu, and Wang (2018) evaluated an XRD analysis of SPI‐based films with varying pine bark extract (PBE) content. They reveal structural changes in the films. Control SPI films display diffraction peaks at 2*θ* values of 8.5° and 19.6°, representing α‐helix and β‐sheet structures, respectively. As PBE concentration increases, the intensity of the 19.6° peak decreases, indicating reduced structural order due to hydrogen bonding interactions between phenolic hydroxyl groups in PBE and amino groups in SPI. Interestingly, films with higher PBE levels show slightly more intense peaks at 19.6°, likely due to excess amorphous PBE. These findings align with SEM observations (Han, Yu, and Wang 2018).

**FIGURE 3:**
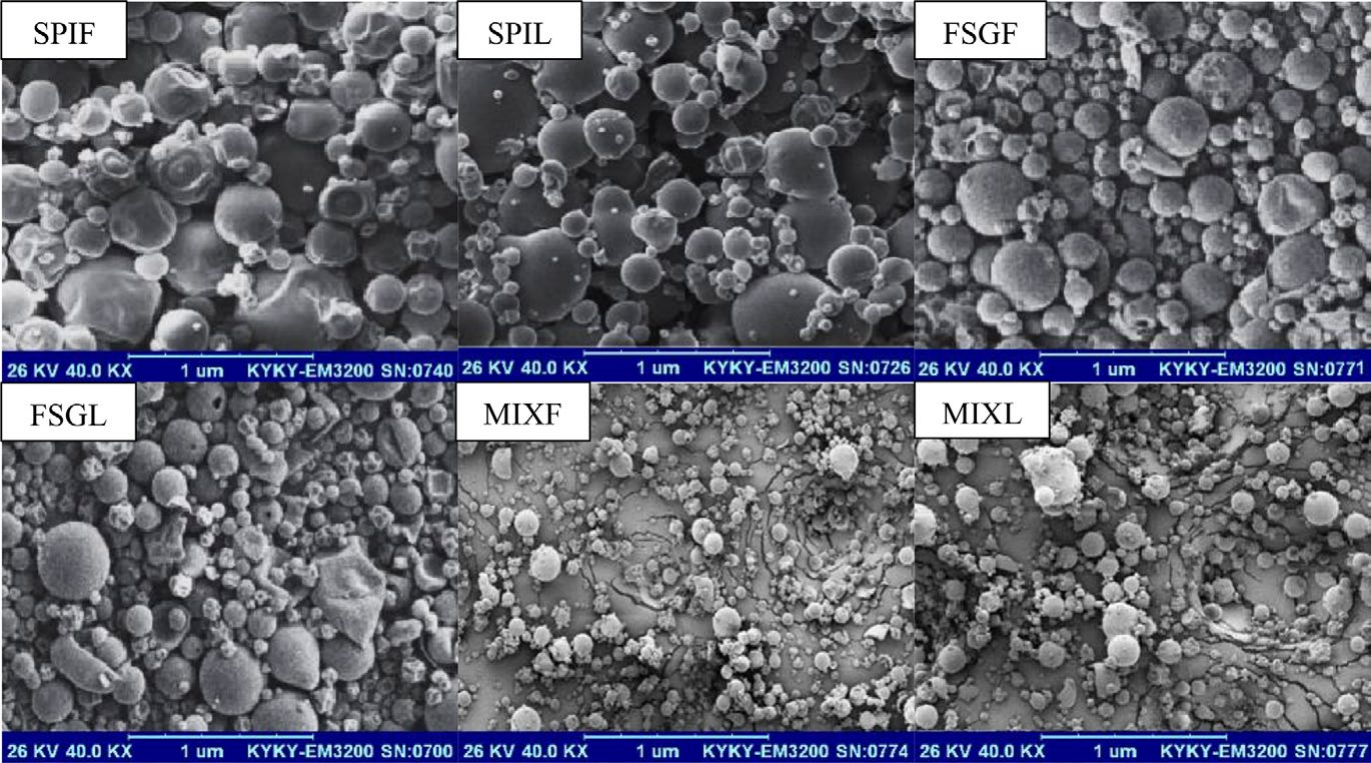
Surface electron microscopy images of different nano‐coating samples. SPIF and SPIL = flower and leaf extract stabilized with soy protein isolate. FSGF and FSGL = flower and leaf extract stabilized with Fenugreek seed gum. MIXF and MIXL = flower and leaf extract stabilized with composition of soy protein isolate and Fenugreek seed gum.

The SEM images in Figure 3 depict the *P. graveolens* extract encapsulated with SPI, FSG, and SPI/FSG. Notably, when a combination of SPI and FSG was utilized, the nanocapsules exhibited a smoother and more spherical shape, devoid of pores, and smaller in size. Conversely, nanocapsules formed solely with SPI displayed slight shrinkage, along with surface dents and pores, which can be attributed to protein denaturation during the vacuum drying process. Choi and Chang (2018) observed similar results, reporting nanocapsules with smooth surfaces when employing a protein/polysaccharide complex as the wall material (Choi and Chang 2018). Esfanjani et al. (2015) also found that microcapsules coated with a combination of whey protein concentrate and pectin exhibited fewer surface dents and pores compared to those coated with whey protein concentrate alone. These findings align with SEM images reported by other researchers who investigated the encapsulation of extracts using protein/polysaccharide wall materials (Kenari et al. 2020; Razavi and Kenari 2021; Yazdan‐Bakhsh et al. 2021).

### 3.6 Properties of Meat Samples

**FIGURE 4:**
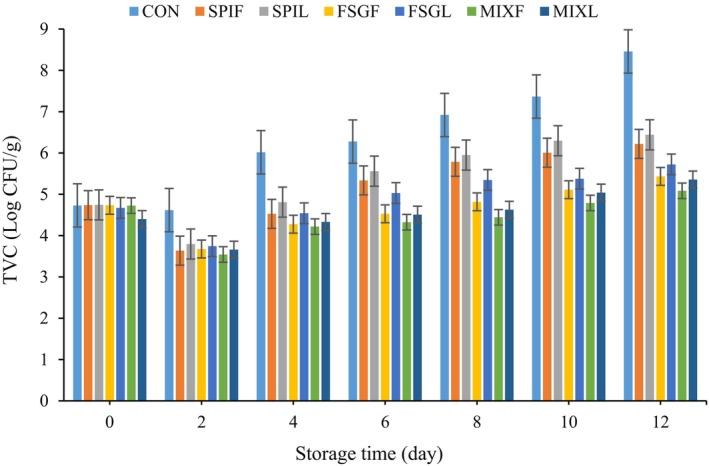
Change in total viable count of bacteria (TVC) in different samples. CON = sample without coating, SPIF, SPIL, FSGF, FSGL, MIXF, and MIXL = sample coated with SPIF, SPIL, FSGF, FSGL, MIXF, and MIXL coatings, respectively.

The quality of meat tends to deteriorate during storage due to spoilage, which can be assessed by analyzing several microbial and chemical parameters such as TVC, pH, TBA, and so on. The primary cause of meat product spoilage is attributed to the rapid proliferation of microorganisms. Meat is typically deemed unfit for consumption once the total viable counts in meat products surpass 106 CFU g^−1^ (Mohammadi, Kamkar, and Misaghi 2018). The impact of various nano‐coatings on the TVC of mutton meat stored at 4°C for a duration of 12 days was illustrated in Figure 4. In comparison to the control group (CON), the mutton samples coated with different types of nano‐coatings exhibited a noteworthy decrease in TVC during storage at 4°C (*p* < 0.05) on day 2. Furthermore, the TVC values of all samples displayed a significant rise as the storage time increased (*p* < 0.05) which is in accordance with the results of other researchers (Song et al. 2020).

The control sample surpassed the threshold for TVC on day 4, whereas the sample coated with SPI coating exceeded the threshold on day 10. The application of the coating acted as a protective shield, effectively separating the meat from its environment. Consequently, this barrier hindered the transfer of essential nutrients into the microbial cells (Zhao et al. 2022). Also, this discrepancy in timing can be attributed to the presence of bioactive compounds, particularly polyphenols, in the coated samples. The inclusion of polyphenols in the coated samples may have contributed to this difference, as previous research has highlighted the antioxidant and antibacterial properties of geranium polyphenols (El Aanachi et al. 2020). Geranium polyphenols exhibit a wide‐ranging antibacterial activity, effectively inhibiting the growth of both Gram‐positive and Gram‐negative bacteria. Several studies reported that geranium polyphenols have the potential to induce morphological alterations in microorganisms, disrupt the bacterial cell wall, and influence the formation of biofilm (Boukhatem, Kameli, and Saidi 2013; Bouzenna and Krichen 2013; Hsouna and Hamdi 2012).

These results are in agreement with those reported by Zhou et al. (2021). They used the encapsulated camellia oil in carrageenan and glucomannan coating to increase the quality of chicken meat. Their findings indicated that the coating led to a significant reduction in microbial counts (*p* < 0.05) compared to samples without coating. The results obtained demonstrated that the coating containing camellia oil effectively prolonged the shelf life of chicken meat by inhibiting microbial growth (Zhou et al. 2021).

**FIGURE 5:**
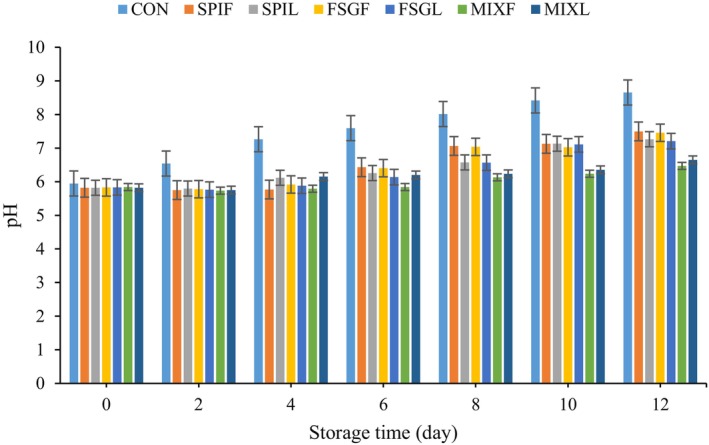
Change in pH of different samples. CON = sample without coating, SPIF, SPIL, FSGF, FSGL, MIXF, and MIXL = sample coated with SPIF, SPIL, FSGF, FSGL, MIXF, and MIXL coatings, respectively.

In general, the pH level is commonly used to assess the freshness of meat. The influence of GE extract on pH was demonstrated in Figure 5 when the biopolymeric‐based coating was incorporated with various types of extract. According to Figure 5, the pH value of the mutton meat samples initially decreased and then increased, which aligns with the results of Zhao et al. (2022). Within the first 0–2 days, the meat transitioned from aerobic respiration to anaerobic respiration, resulting in the production of lactic acid and phosphoric acid. As a result, the meat was not completely rid of acid, leading to a decrease in pH value during the initial stage (Xiong, Chen et al. 2020; Xiong, Li et al. 2020). However, after 4 days of storage the pH value of the control meat exhibited a rapid increase, surpassing 7.59 after 6 days, indicating a state of meat deterioration. This rise in pH can be attributed to the bacterial activity on the meat surface during the early stages of storage, which leads to the release of basic amines. The pH values of the samples coated with a composite coating containing flower extract were lower compared to the individual coatings and the control group (CON). This difference can be attributed to the presence of antioxidant and antibacterial components in the coating, such as phenolic compounds. These components inhibit microbial growth and hold up lipid and protein oxidation, resulting in a lower pH value.

Food pH is an indicator of food safety and quality and red meats should have a pH level below 7–7.5 to ensure consumer safety. All samples coated with nano‐coating were in the standard range in terms of pH value. These findings underscore the importance of monitoring and maintaining appropriate pH levels in food products to safeguard public health (Jooyandeh et al. 2023). Research findings indicate that microcapsules enveloped by a combination of protein and polysaccharide tend to exhibit greater resistance to variations in pH levels, increased ionic strength, and elevated temperatures when compared to microcapsules coated solely with either protein or polysaccharide (Choi and Chang 2018). These results are in agreement with previous studies for mutton meat coated with *Cordia* coating containing *R. officinalis* essential oil (Jooyandeh et al. 2023), and *L. sativum* seed mucilage and *S. hortensis* essential oil in lamb meat (Farahani et al. 2024).

**FIGURE 6:**
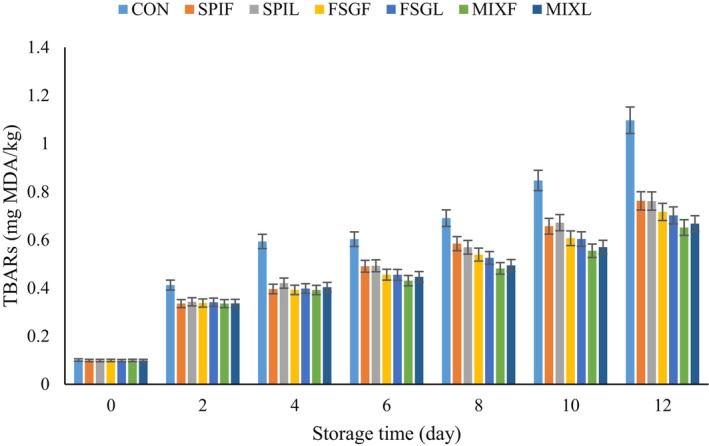
Change in thiobarbituric acid (TBARs) content of different samples. CON = sample without coating, SPIF, SPIL, FSGF, FSGL, MIXF, and MIXL = sample coated with SPIF, SPIL, FSGF, FSGL, MIXF, and MIXL coatings, respectively.

TBA value is a common index to evaluate lipid oxidation in mutton meat. Figure 6 depicts the changes in TBA values for both uncoated and coated samples. When the TBA values exceeded 0.5 mg kg^−1^ in mutton meat, consumers might detect an undesirable off‐flavor. The data presented in Figure 6 demonstrated a consistent increasing trend across all samples. The initial TBA of fresh mutton samples was recorded at 0.207 mg kg^−1^. At day 4 of storage time, the uncoated sample surpassed the 0.5 mg kg^−1^ threshold, reaching 0.59 mg kg^−1^ by the tenth day. This result indicates a significant level of lipid oxidation in the control sample. However, the extract loaded in coatings had a notable effect in delaying the production of secondary lipid oxidation products. The complex coating, in particular, acted as an impressive barrier, preventing the reaction between oxygen and meat. As a result, the TBA value of the coated samples was significantly lower than that of the control sample samples (*p* < 0.05).

This outcome could be attributed to the presence of bioactive compounds in the coating. Actually, these compounds by scavenging free radicals, and chelating metal ions inhibit lipid peroxidation and regulate the levels of antioxidant enzymes. Additionally, the nano‐coating played a crucial role in impeding the lipid oxidation process by preventing direct contact between oxygen and the meat samples, allowing the oil component in the nano‐coating to react first. Zhou et al. (2021) stated that the application of polyphenols in coating resulted in a lower TBA value in the chicken meat samples due to a decrease in the lipid oxidation of fats or proteins. This result corresponded with microbial count (Zhou et al. 2021). Different studies have shown comparable outcomes when using various types of coatings on other types of meat. For instance, Raeisi et al. (2014) evaluated the effect of *Zataria multiflora* and grape seed extract in carboxymethyl cellulose coating on the inhibition of lipid oxidation in rainbow trout meat and observed similar results. Similarly, Jridi et al. (2018) reported similar results when using a gelatin coating incorporated with henna extract on beef. Khare et al. (2016) also observed comparable outcomes with an edible coating based on carrageenan and cinnamon oil on chicken fillet (Khare et al. 2016). Another study by Javan et al. (2024) demonstrated that ZnO nanoparticles in *Vicia villosa* protein isolate had similar effects on chicken breast meat. These findings highlight the potential of different coatings to enhance the quality and shelf life of various types of meat (Javan et al. 2024). The change in the visual image of samples during storage is illustrated in Figure S1.

## 4 Conclusion

This study aimed to create a sustainable nano‐coating material by incorporating geranium extract into SPI and FSG biopolymers. The resulting green nano‐coating material exhibited excellent performance and was subsequently utilized to extend the shelf life of fresh mutton meat. The findings demonstrated that the application of coatings had a significant impact on the growth of microorganisms in meat, resulting in a reduction of total viable counts to below 106 CFU g^−1^. Moreover, the coatings were found to effectively inhibit lipid oxidation in fresh meat, thereby preserving its quality. As a result of these effects, the shelf life of fresh meat was extended from 4 to 12 days. Based on the outcomes, it was deduced that coatings composed of SPI/FSG with 2000 ppm of flower geranium extract could efficiently enhance the shelf life of fresh meat. The utilization of this geranium extract protein/carbohydrate nano‐coating presents a promising approach in the development of edible packaging materials that can effectively prolong the shelf life of fresh meat, offering potential benefits for the food industry.

## Author Contributions

**Farzad Ebrahimi:** conceptualization (equal), data curation (equal), formal analysis (equal), investigation (equal), methodology (equal), writing – original draft (equal). **Nader Habibi:** software (equal), supervision (equal), validation (equal), writing – original draft (equal), writing – review and editing (equal). **Mohammadyar Hosseini:** data curation (equal), methodology (equal), project administration (equal), validation (equal), writing – original draft (equal), writing – review and editing (equal).

## Ethics Statement

This article does not contain any studies with human or animal subjects.

## Conflicts of Interest

The authors declare no conflicts of interest.

## Supporting information


Figure S1

## Data Availability

Data will be made available on request.

## References

[fsn34618-bib-0001] Abdelbaky, A. S. , T. A. Abd El‐Mageed , A. O. Babalghith , S. Selim , and A. M. Mohamed . 2022. “Green Synthesis and Characterization of ZnO Nanoparticles Using *Pelargonium odoratissimum* (L.) Aqueous Leaf Extract and Their Antioxidant, Antibacterial and Anti‐Inflammatory Activities.” Antioxidants 11, no. 8: 1444. 35892646 10.3390/antiox11081444 PMC9329751

[fsn34618-bib-0002] Ahmad, N. , F. Anwar , S. Hameed , and M. C. Boyce . 2011. “Antioxidant and Antimicrobial Attributes of Different Solvent Extracts From Leaves and Flowers of Akk [*Calotropis procera* (Ait.) (Ait. F.)].” Journal of Medicinal Plant Research 5, no. 19: 4879–4887.

[fsn34618-bib-0003] Ahmadian, S. , R. E. Kenari , Z. R. Amiri , F. Sohbatzadeh , and M. H. H. Khodaparast . 2024. “Fabrication of Double Nano‐Emulsions Loaded With Hyssop ( *Hyssopus officinalis* L.) Extract Stabilized With Soy Protein Isolate Alone and Combined With Chia Seed Gum in Controlling the Oxidative Stability of Canola Oil.” Food Chemistry 430: 137093. 37562266 10.1016/j.foodchem.2023.137093

[fsn34618-bib-0004] Ahmed, G. H. G. , A. Fernández‐González , and M. E. D. García . 2020. “Nano‐Encapsulation of Grape and Apple Pomace Phenolic Extract in Chitosan and Soy Protein via Nanoemulsification.” Food Hydrocolloids 108: 105806.

[fsn34618-bib-0005] Al‐Shammari, B. , R. Al‐Ali , and A. Al‐Sahi . 2019. “Electrical, Characterization and Functional Properties of Extract Gum (*Tniqonella foenum graecum* L.) From Fenugreek Seeds.” Paper Presented at the IOP Conference Series: Earth and Environmental Science.

[fsn34618-bib-0006] Amiri, R. , A. Nikbakht , and N. Etemadi . 2015. “Alleviation of Drought Stress on Rose Geranium [ *Pelargonium graveolens* (L.) Herit.] in Terms of Antioxidant Activity and Secondary Metabolites by Mycorrhizal Inoculation.” Scientia Horticulturae 197: 373–380.

[fsn34618-bib-0007] Bennett, S. D. , K. A. Walsh , and L. H. Gould . 2013. “Foodborne Disease Outbreaks Caused by *Bacillus cereus* , *Clostridium perfringens*, and *Staphylococcus aureus* —United States, 1998–2008.” Clinical Infectious Diseases 57, no. 3: 425–433. 23592829 10.1093/cid/cit244 PMC11334977

[fsn34618-bib-0008] Boukhatem, M. N. , A. Kameli , and F. Saidi . 2013. “Essential Oil of Algerian Rose‐Scented Geranium ( *Pelargonium graveolens* ): Chemical Composition and Antimicrobial Activity Against Food Spoilage Pathogens.” Food Control 34, no. 1: 208–213.

[fsn34618-bib-0009] Boukhris, M. , M. S. Simmonds , S. Sayadi , and M. Bouaziz . 2013. “Chemical Composition and Biological Activities of Polar Extracts and Essential Oil of Rose‐Scented Geranium, *Pelargonium graveolens* .” Phytotherapy Research 27, no. 8: 1206–1213. 23027699 10.1002/ptr.4853

[fsn34618-bib-0010] Bouzenna, H. , and L. Krichen . 2013. “ *Pelargonium graveolens* L'Her. and *Artemisia arborescens* L. Essential Oils: Chemical Composition, Antifungal Activity Against *Rhizoctonia solani* and Insecticidal Activity Against *Rhysopertha dominica* .” Natural Product Research 27, no. 9: 841–846. 22840199 10.1080/14786419.2012.711325

[fsn34618-bib-0011] Carrapiso, A. I. , M. Pimienta , L. Martín , V. Cardenia , and A. I. Andrés . 2023. “Effect of a Chitosan Coating Enriched With an Olive Leaf Extract on the Characteristics of Pork Burgers.” Food 12, no. 20: 3757. 10.3390/foods12203757 PMC10606866 37893650

[fsn34618-bib-0012] Choi, Y.‐R. , and Y. H. Chang . 2018. “Microencapsulation of Gallic Acid Through the Complex of Whey Protein Concentrate‐Pectic Polysaccharide Extracted From *Ulmus davidiana* .” Food Hydrocolloids 85: 222–228.

[fsn34618-bib-0013] Dash, D. K. , R. K. Panik , A. K. Sahu , and V. Tripathi . 2020. “Role of Nanobiotechnology in Drug Discovery, Development and Molecular Diagnostic.” In Applications of Nanobiotechnology. Lower Thames Street, London: IntechOpen.

[fsn34618-bib-0014] Dimitrova, M. , D. Mihaylova , A. Popova , J. Alexieva , T. Sapundzhieva , and H. Fidan . 2015. “Phenolic Profile, Antibacterial and Antioxidant Activity of *Pelargonium graveolens* leaves' Extracts.” Scientific Bulletin. Series F. Biotechnologies 7: 130–136.

[fsn34618-bib-0015] Dong, D. , and B. Cui . 2021. “Fabrication, Characterization and Emulsifying Properties of Potato Starch/Soy Protein Complexes in Acidic Conditions.” Food Hydrocolloids 115: 106600.

[fsn34618-bib-0016] Ebrahimian, P. , A. Najafi , and A. Abedinia . 2024. “Effect of Nanoencapsulated Pistachio Green Hull Extract in the Carboxymethyl Cellulose and Soy Protein Isolate Edible Coatings on Shelf‐Life Quality of Fresh Pistachio.” Journal of Food Processing and Preservation 2024, no. 1: 5524814.

[fsn34618-bib-0017] Economou, V. , A. Tsitsos , A. Theodoridis , I. Ambrosiadis , and G. Arsenos . 2022. “Effects of Chitosan Coatings on Controlling Listeria Monocytogenes and Methicillin‐Resistant *Staphylococcus aureus* in Beef and Mutton Cuts.” Applied Sciences 12, no. 22: 11345.

[fsn34618-bib-0018] El Aanachi, S. , L. Gali , S. N. Nacer , C. Bensouici , K. Dari , and H. Aassila . 2020. “Phenolic Contents and In Vitro Investigation of the Antioxidant, Enzyme Inhibitory, Photoprotective, and Antimicrobial Effects of the Organic Extracts of *Pelargonium graveolens* Growing in Morocco.” Biocatalysis and Agricultural Biotechnology 29: 101819.

[fsn34618-bib-0019] Ennaifer, M. , T. Bouzaiene , C. Messaoud , and M. Hamdi . 2020. “Phytochemicals, Antioxidant, Anti‐Acetyl‐Cholinesterase, and Antimicrobial Activities of Decoction and Infusion of *Pelargonium graveolens* .” Natural Product Research 34, no. 18: 2634–2638. 30584784 10.1080/14786419.2018.1547299

[fsn34618-bib-0020] Esfanjani, A. F. , S. M. Jafari , E. Assadpoor , and A. Mohammadi . 2015. “Nano‐Encapsulation of Saffron Extract Through Double‐Layered Multiple Emulsions of Pectin and Whey Protein Concentrate.” Journal of Food Engineering 165: 149–155.

[fsn34618-bib-0021] Esmaeilzadeh Kenari, R. , and R. Razavi . 2022. “Phenolic Profile and Antioxidant Activity of Free/Bound Phenolic Compounds of Sesame and Properties of Encapsulated Nanoparticles in Different Wall Materials.” Food Science & Nutrition 10: 525–535. 35154689 10.1002/fsn3.2712 PMC8825734

[fsn34618-bib-0022] Farahani, M. , F. Shahidi , F. T. Yazdi , and A. Ghaderi . 2024. “Antimicrobial and Antioxidant Effects of an Edible Coating of *Lepidium sativum* Seed Mucilage and *Satureja hortensis* L. Essential Oil in Uncooked Lamb Meat.” Food Control 158: 110240.

[fsn34618-bib-0023] Fatemi, A. , A. Najafi , R. Razavi , and S. Jafarzadeh . 2024. “Characterizing the Antioxidant and Antifungal Properties of Nano‐Encapsulated Pistachio Hull Extract in Fenugreek Seed Gum to Maintain the Quality and Safety of Fresh Pistachio.” Food Science & Nutrition 12: 5561–5571. 39139972 10.1002/fsn3.4209 PMC11317734

[fsn34618-bib-0024] González, N. , M. Marquès , M. Nadal , and J. L. Domingo . 2020. “Meat Consumption: Which Are the Current Global Risks? A Review of Recent (2010–2020) Evidences.” Food Research International 137: 109341. 33233049 10.1016/j.foodres.2020.109341 PMC7256495

[fsn34618-bib-0025] Gorzin, M. , M. Saeidi , S. Javidi , E.‐K. Seow , and A. Abedinia . 2024. “Nanoencapsulation of Oliveria Decumbens Vent./Basil Essential Oils Into Gum Arabic/Maltodextrin: Improved In Vitro Bioaccessibility and Minced Beef Meat Safety.” International Journal of Biological Macromolecules 270: 132288. 38735604 10.1016/j.ijbiomac.2024.132288

[fsn34618-bib-0026] Guerrero, P. , M. G. O'Sullivan , J. P. Kerry , and K. de la Caba . 2015. “Application of Soy Protein Coatings and Their Effect on the Quality and Shelf‐Life Stability of Beef Patties.” RSC Advances 5, no. 11: 8182–8189.

[fsn34618-bib-0027] Guo, Q. , B. Cui , C. Yuan , et al. 2024. “Fabrication of Dry S/O/W Microcapsule and Its Probiotic Protection Against Different Stresses.” Journal of the Science of Food and Agriculture 104, no. 5: 2842–2850. 38012057 10.1002/jsfa.13175

[fsn34618-bib-0028] Hajian‐Tilaki, A. , R. E. Kenari , R. Razavi , and R. Farahmandfar . 2024. “Phenolic Profile, Antioxidant Properties, and Pollen Spectra of Iranian‐Originated Honeys.” European Food Research and Technology 250: 1–13.

[fsn34618-bib-0029] Han, Y. , M. Yu , and L. Wang . 2018. “Bio‐Based Films Prepared With Soybean By‐Products and Pine ( *Pinus densiflora* ) Bark Extract.” Journal of Cleaner Production 187: 1–8.

[fsn34618-bib-0030] Harzallah, A. A. , K. Hachama , and A. Khadraoui . 2022. “Polyphenol Analysis by HPLC‐DAD, and Antimicrobial and Antioxidant Activities of Two Species Extracts of Pelargonium: *P. graveolens* and *P. zonale* .” Nutrition and Santé 11, no. 1: 48–56.

[fsn34618-bib-0031] Hsouna, A. B. , and N. Hamdi . 2012. “Phytochemical Composition and Antimicrobial Activities of the Essential Oils and Organic Extracts From *Pelargonium graveolens* Growing in Tunisia.” Lipids in Health and Disease 11: 1–7. 23216669 10.1186/1476-511X-11-167 PMC3539951

[fsn34618-bib-0032] Jafari, S. Z. , S. Jafarian , M. Hojjati , and L. Najafian . 2022. “Evaluation of Antioxidant Activity of Nano‐and Microencapsulated Rosemary ( *Rosmarinus officinalis* L.) Leaves Extract in Cress ( *Lepidium sativum* ) and Basil ( *Ocimum basilicum* ) Seed Gums for Enhancing Oxidative Stability of Sunflower Oil.” Food Science & Nutrition 10, no. 6: 2111–2119. 35702297 10.1002/fsn3.2827 PMC9179134

[fsn34618-bib-0033] Javan, A. J. , S. Baktash , B. S. Yancheshmeh , et al. 2024. “Effect of *Vicia villosa* Protein Isolate‐Based Edible Coating Incorporated With ZnO Nanoparticles on the Shelf‐Life of Chicken Breast Meat During Cold Storage.” Food Hydrocolloids for Health 5: 100176.

[fsn34618-bib-0034] Jooyandeh, H. , M. Ebrahimi Hemmati Kaykha , B. Alizadeh Behbahani , and M. Noshad . 2023. “Evaluating the Quality of Mutton Meat Coated With *Cordia myxa* Fruit Mucilage Containing *Rosmarinus officinalis* Essential Oil During Cold Storage.” Journal of Food Measurement and Characterization 17, no. 3: 2062–2074.

[fsn34618-bib-0035] Jridi, M. , L. Mora , N. Souissi , M.‐C. Aristoy , M. Nasri , and F. Toldrá . 2018. “Effects of Active Gelatin Coated With Henna ( *L. inermis* ) Extract on Beef Meat Quality During Chilled Storage.” Food Control 84: 238–245.

[fsn34618-bib-0036] Kenari, R. E. , Z. R. Amiri , A. Motamedzadegan , J. M. Milani , J. Farmani , and R. Farahmandfar . 2020. “Optimization of Iranian Golpar (*Heracleum persicum*) Extract Encapsulation Using Sage (*Salvia macrosiphon*) Seed Gum: Chitosan as a Wall Materials and Its Effect on the Shelf Life of Soybean Oil During Storage.” Journal of Food Measurement and Characterization 14: 2828–2839.

[fsn34618-bib-0037] Kenari, R. E. , and R. Razavi . 2022. “Encapsulation of Bougainvillea ( *Bougainvillea spectabilis* ) Flower Extract in *Urtica dioica* L. Seed Gum: Characterization, Antioxidant/Antimicrobial Properties, and In Vitro Digestion.” Food Science & Nutrition 10, no. 10: 3436–3443. 36249979 10.1002/fsn3.2944 PMC9548349

[fsn34618-bib-0038] Khan, I. , K. Saeed , and I. Khan . 2019. “Nanoparticles: Properties, Applications and Toxicities.” Arabian Journal of Chemistry 12, no. 7: 908–931.

[fsn34618-bib-0039] Khare, A. K. , R. J. Abraham , V. A. Rao , and R. N. Babu . 2016. “Utilization of Carrageenan, Citric Acid and Cinnamon Oil as an Edible Coating of Chicken Fillets to Prolong Its Shelf Life Under Refrigeration Conditions.” Veterinary World 9, no. 2: 166–175. 27051203 10.14202/vetworld.2016.166-175 PMC4819367

[fsn34618-bib-0040] Kumar, M. U. , and M. K. Babu . 2014. “Design and Evaluation of Fast Dissolving Tablets Containing Diclofenac Sodium Using Fenugreek Gum as a Natural Superdisintegrant.” Asian Pacific Journal of Tropical Biomedicine 4: S329–S334. 25183106 10.12980/APJTB.4.2014B672 PMC4025345

[fsn34618-bib-0041] Langroodi, A. M. , H. Tajik , T. Mehdizadeh , M. Moradi , E. M. Kia , and A. Mahmoudian . 2018. “Effects of Sumac Extract Dipping and Chitosan Coating Enriched With Zataria Multiflora Boiss Oil on the Shelf‐Life of Meat in Modified Atmosphere Packaging.” LWT–Food Science and Technology 98: 372–380.

[fsn34618-bib-0042] Mansour, M. , M. Salah , and X. Xu . 2020. “Effect of Microencapsulation Using Soy Protein Isolate and Gum Arabic as Wall Material on Red Raspberry Anthocyanin Stability, Characterization, and Simulated Gastrointestinal Conditions.” Ultrasonics Sonochemistry 63: 104927. 31952001 10.1016/j.ultsonch.2019.104927

[fsn34618-bib-0043] Mohammadi, H. , A. Kamkar , and A. Misaghi . 2018. “Nanocomposite Films Based on CMC, Okra Mucilage and ZnO Nanoparticles: Physico Mechanical and Antibacterial Properties.” Carbohydrate Polymers 181: 351–357. 29253983 10.1016/j.carbpol.2017.10.045

[fsn34618-bib-0044] Nilavukkarasi, M. , S. Vijayakumar , and S. Prathipkumar . 2020. “Capparis Zeylanica Mediated Bio‐Synthesized ZnO Nanoparticles as Antimicrobial, Photocatalytic and Anti‐Cancer Applications.” Materials Science for Energy Technologies 3: 335–343.

[fsn34618-bib-0045] Odeyemi, O. A. , O. O. Alegbeleye , M. Strateva , and D. Stratev . 2020. “Understanding Spoilage Microbial Community and Spoilage Mechanisms in Foods of Animal Origin.” Comprehensive Reviews in Food Science and Food Safety 19, no. 2: 311–331. 33325162 10.1111/1541-4337.12526

[fsn34618-bib-0046] Oliveira, F. M. , R. M. Oliveira , L. T. G. Buchweitz , et al. 2022. “Encapsulation of Olive Leaf Extract ( *Olea europaea* L.) in Gelatin/Tragacanth Gum by Complex Coacervation for Application in Sheep Meat Hamburger.” Food Control 131: 108426.

[fsn34618-bib-0047] Patil, M. P. , and G.‐D. Kim . 2017. “Eco‐Friendly Approach for Nanoparticles Synthesis and Mechanism Behind Antibacterial Activity of Silver and Anticancer Activity of Gold Nanoparticles.” Applied Microbiology and Biotechnology 101: 79–92. 27915376 10.1007/s00253-016-8012-8

[fsn34618-bib-0048] Pethick, D. , J.‐F. Hocquette , N. Scollan , and F. Dunshea . 2021. “Improving the Nutritional, Sensory and Market Value of Meat Products From Sheep and Cattle.” Animal 15: 100356. 34600858 10.1016/j.animal.2021.100356

[fsn34618-bib-0049] Raeisi, M. , H. Tajik , J. Aliakbarlu , and S. Valipour . 2014. “Effect of Carboxymethyl Cellulose Edible Coating Containing Zataria Multiflora Essential Oil and Grape Seed Extract on Chemical Attributes of Rainbow Trout Meat.” Paper Presented at the Veterinary Research Forum: An International Quarterly Journal. PMC4279636 25568700

[fsn34618-bib-0050] Razavi, R. , and R. E. Kenari . 2021. “Antioxidant Evaluation of *Fumaria parviflora* L. Extract Loaded Nanocapsules Obtained by Green Extraction Methods in Oxidative Stability of Sunflower Oil.” Journal of Food Measurement and Characterization 15, no. 3: 2448–2457.

[fsn34618-bib-0051] Razavi, R. , R. E. Kenari , J. Farmani , and M. Jahanshahi . 2020. “Fabrication of Zein/Alginate Delivery System for Nanofood Model Based on Pumpkin.” International Journal of Biological Macromolecules 165: 3123–3134. 33127546 10.1016/j.ijbiomac.2020.10.176

[fsn34618-bib-0052] Razavi, R. , Y. Maghsoudlou , M. Aalami , and M. Ghorbani . 2021. “Impact of Carboxymethyl Cellulose Coating Enriched With *Thymus vulgaris* L. Extract on Physicochemical, Microbial, and Sensorial Properties of Fresh Hazelnut ( *Corylus avellana* L.) During Storage.” Journal of Food Processing and Preservation 45, no. 4: e15313.

[fsn34618-bib-0053] Sarvinehbaghi, M. B. , M. Ahmadi , M. Shiran , and M. Azizkhani . 2021. “Antioxidant and Antimicrobial Activity of Red Onion ( *Allium cepa* , L.) Extract Nanoencapsulated in Native Seed Gums Coating and Its Effect on Shelf‐Life Extension of Beef Fillet.” Journal of Food Measurement and Characterization 15, no. 5: 4771–4780.

[fsn34618-bib-0054] Shukla, A. K. , A. Yadav , R. Vishwakarma , and S. Mishra . 2020. “Applications, Isolation and Characterization of Fenugreek Seed Gum as Pharmaceutical Excipient.” Journal of Medical Pharmaceutical and Allied Sciences 9, no. 2: 920–2491.

[fsn34618-bib-0055] Song, D.‐H. , V. B. Hoa , H. W. Kim , et al. 2021. “Edible Films on Meat and Meat Products.” Coatings 11, no. 11: 1344.

[fsn34618-bib-0056] Song, W. , Y. Du , C. Yang , et al. 2020. “Development of PVA/EVA‐Based Bilayer Active Film and Its Application to Mutton.” LWT—Food Science and Technology 133: 110109.

[fsn34618-bib-0057] Tavares, L. , and C. P. Z. Noreña . 2020. “Encapsulation of Ginger Essential Oil Using Complex Coacervation Method: Coacervate Formation, Rheological Property, and Physicochemical Characterization.” Food and Bioprocess Technology 13: 1405–1420.

[fsn34618-bib-0058] Timilsena, Y. P. , R. Adhikari , S. Kasapis , and B. Adhikari . 2016. “Molecular and Functional Characteristics of Purified Gum From Australian Chia Seeds.” Carbohydrate Polymers 136: 128–136. 26572338 10.1016/j.carbpol.2015.09.035

[fsn34618-bib-0059] Tsitsos, A. , V. Economou , G. Arsenos , T. Kalitsis , A. Argyriadou , and A. Theodoridis . 2021. “Greek and European Consumer Behaviour Towards Beef, Lamb and Mutton Meat Safety and Quality: A Review.” International Journal of Agricultural Resources, Governance and Ecology 17, no. 2–4: 414–431.

[fsn34618-bib-0060] Tsitsos, A. , V. Economou , E. Chouliara , G. Koutouzidou , G. Arsenos , and I. Ambrosiadis . 2023. “Effect of Chitosan and Alginate‐Based Edible Membranes With Oregano Essential Oil and Olive Oil in the Microbiological, Physicochemical and Organoleptic Characteristics of Mutton.” Microorganisms 11, no. 2: 507. 36838470 10.3390/microorganisms11020507 PMC9961988

[fsn34618-bib-0061] Xiong, Y. , M. Chen , R. D. Warner , and Z. Fang . 2020. “Incorporating Nisin and Grape Seed Extract in Chitosan‐Gelatine Edible Coating and Its Effect on Cold Storage of Fresh Pork.” Food Control 110: 107018.

[fsn34618-bib-0062] Xiong, Y. , S. Li , R. D. Warner , and Z. Fang . 2020. “Effect of Oregano Essential Oil and Resveratrol Nanoemulsion Loaded Pectin Edible Coating on the Preservation of Pork Loin in Modified Atmosphere Packaging.” Food Control 114: 107226.

[fsn34618-bib-0063] Xu, L. , T. Wang , Y. Shan , R. Wang , and C. Yi . 2024. “Soybean Protein Isolate Inhibiting the Retrogradation of Fresh Rice Noodles: Combined Experimental Analysis and Molecular Dynamics Simulation.” Food Hydrocolloids 151: 109877.

[fsn34618-bib-0064] Xu, Z. , Y. Wu , L. Song , A. Chinnathambi , S. A. Alharbi , and L. Fang . 2020. “Anticarcinogenic Effect of Zinc Oxide Nanoparticles Synthesized From Rhizoma Paridis Saponins on Molt‐4 Leukemia Cells.” Journal of King Saud University, Science 32, no. 3: 1865–1871.

[fsn34618-bib-0065] Yazdan‐Bakhsh, M. , M. Nasr‐Esfahani , R. Esmaeilzadeh‐Kenari , and M. Fazel‐Najafabadi . 2021. “Evaluation of Antioxidant Properties of Heracleum Lasiopetalum Extract in Multilayer Nanoemulsion With Biopolymer Coating to Control Oxidative Stability of Sunflower Oil.” Journal of Food Measurement and Characterization 15, no. 2: 1014–1023.

[fsn34618-bib-0066] Yuan, G. , X. Chen , and D. Li . 2016. “Chitosan Films and Coatings Containing Essential Oils: The Antioxidant and Antimicrobial Activity, and Application in Food Systems.” Food Research International 89: 117–128. 28460897 10.1016/j.foodres.2016.10.004

[fsn34618-bib-0067] Zhao, R. , Y. Zhang , H. Chen , R. Song , and Y. Li . 2022. “Performance of Eugenol Emulsion/Chitosan Edible Coating and Application in Fresh Meat Preservation.” Journal of Food Processing and Preservation 46, no. 3: e16407.

[fsn34618-bib-0068] Zhou, X. , X. Zong , M. Zhang , et al. 2021. “Effect of Konjac Glucomannan/Carrageenan‐Based Edible Emulsion Coatings With Camellia Oil on Quality and Shelf‐Life of Chicken Meat.” International Journal of Biological Macromolecules 183: 331–339. 33930444 10.1016/j.ijbiomac.2021.04.165

