# Supplementary figures and images for: Nano‐Coating Loaded With Leaf and Flowers of Pelargonium graveolens Plant Extract Stabilized With Fenugreek Seed Gum and Soy Protein Isolate in Increasing the Shelf Life of Mutton Fillet
Source: Food Sci Nutr. 2024 Dec 30;13(1):e4618. doi: 10.1002/fsn3.4618 (PMC11717032; doi:10.1002/fsn3.4618)

| Samples | Storage time (day) | | | | | | |
| --- | --- | --- | --- | --- | --- | --- | --- |
| 0 | 2 | 4 | 6 | 8 | 10 | 12 |
| CON | 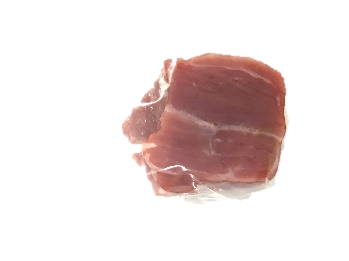 | 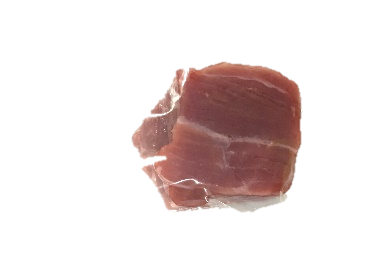 | 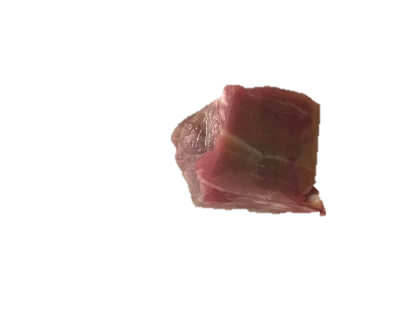 | 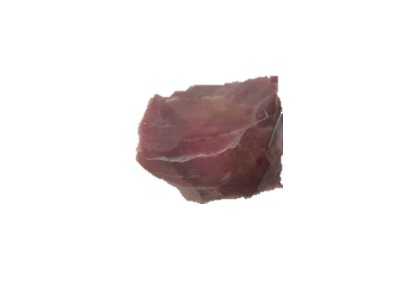 | 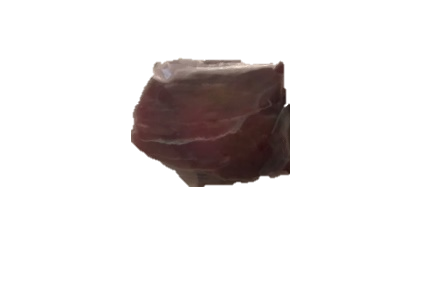 | 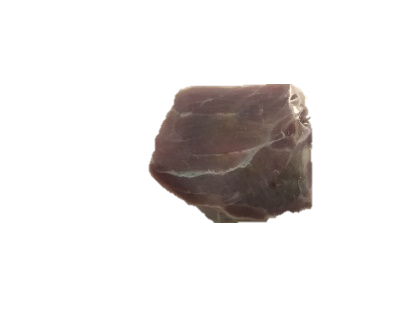 | 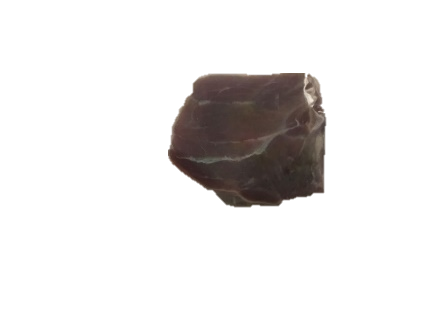 |
| SPIF | 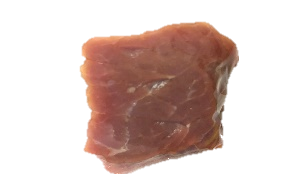 | 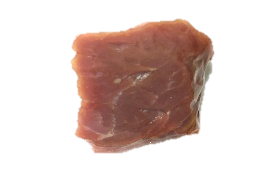 | 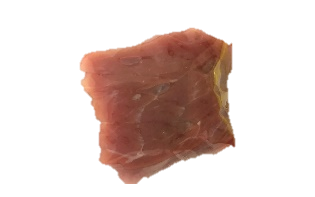 | 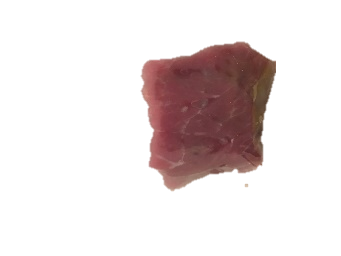 | 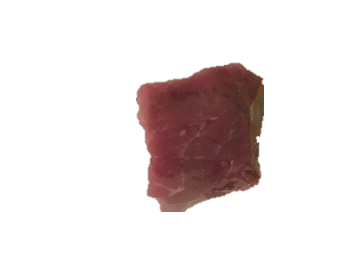 | 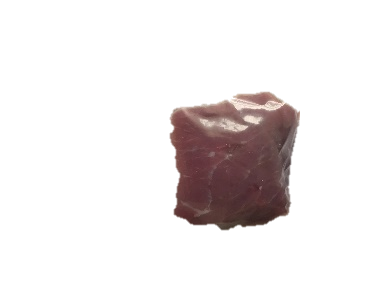 | 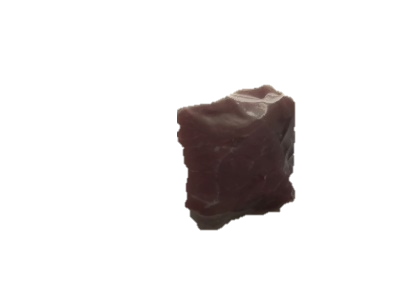 |
| SPIL | 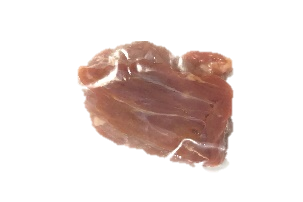 | 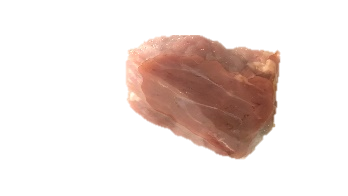 | 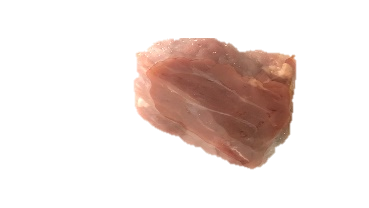 | 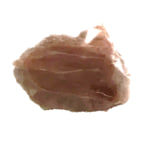 | 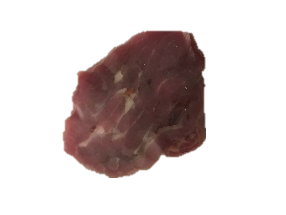 | 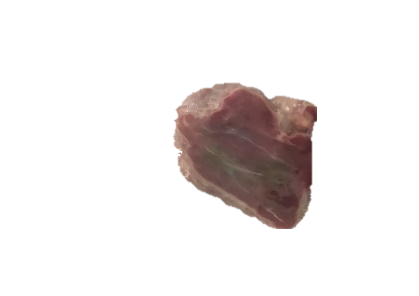 | 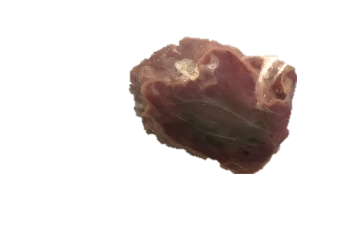 |
| FSGF | 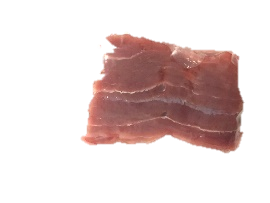 | 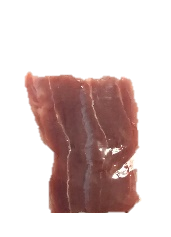 | 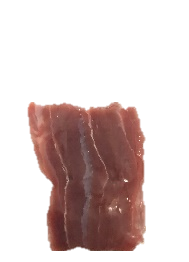 | 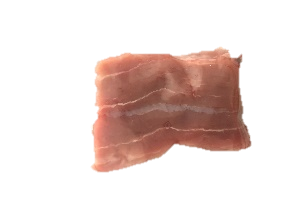 | 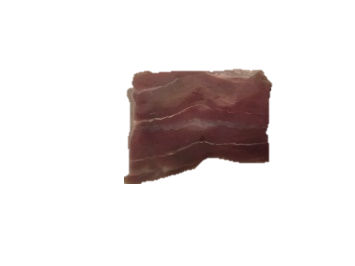 | 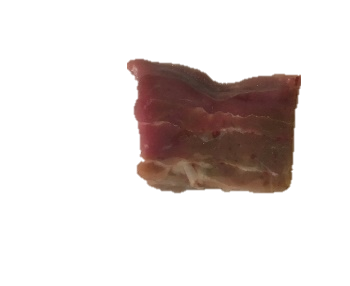 | 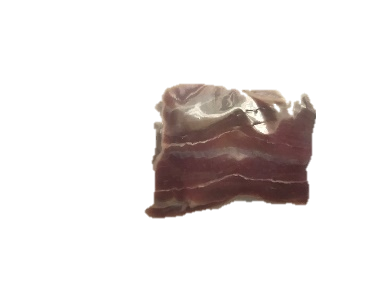 |
| FSGL | 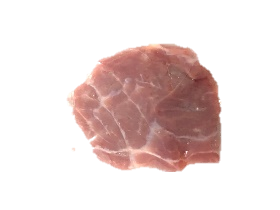 | 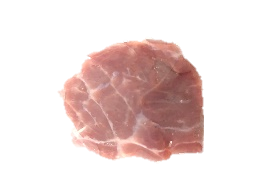 | 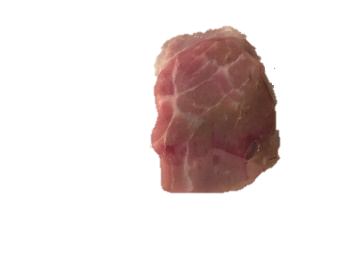 | 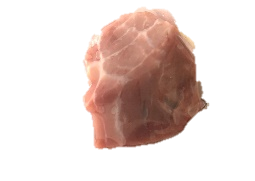 | 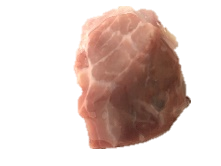 | 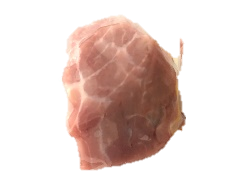 | 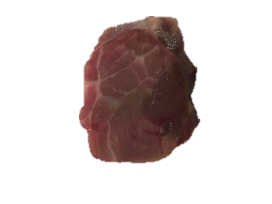 |
| MIXF | 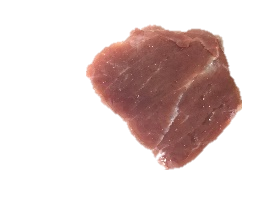 | 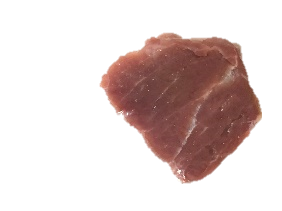 | 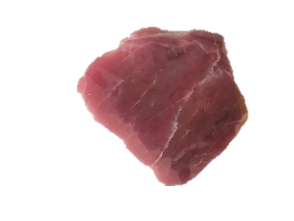 | 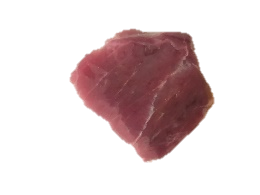 | 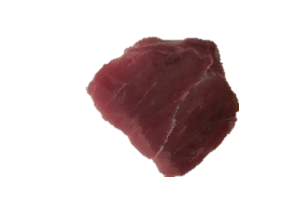 | 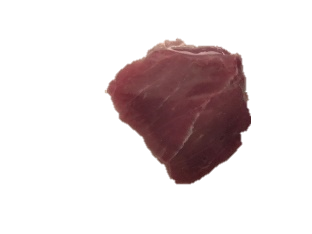 | 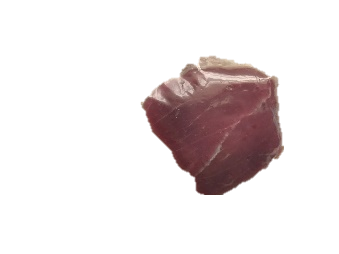 |
| MIXL | 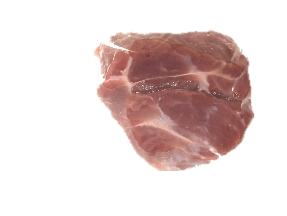 | 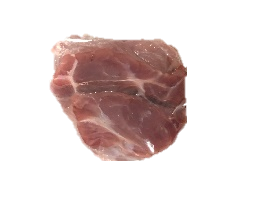 | 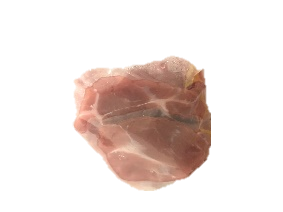 | 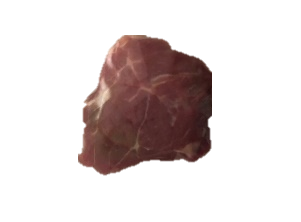 | 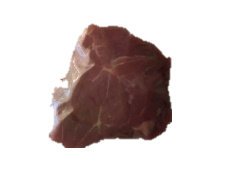 | 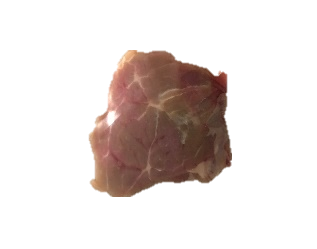 | 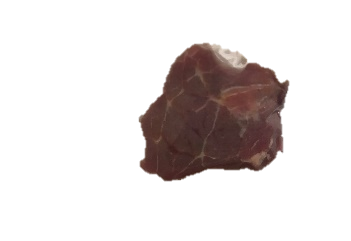 |

Supplement: Supplementary file 1 — Figure S1 [file FSN3-13-e4618-s001.docx]
